# The Limits of Individual Identification from Sample Allele Frequencies: Theory and Statistical Analysis

**DOI:** 10.1371/journal.pgen.1000628

**Published:** 2009-10-02

**Authors:** Peter M. Visscher, William G. Hill

**Affiliations:** 1 Queensland Institute of Medical Research, Brisbane, Australia; 2 Institute of Evolutionary Biology, School of Biological Sciences, University of Edinburgh, United Kingdom; The University of Queensland, Australia

## Abstract

It was shown recently using experimental data that it is possible under certain conditions to determine whether a person with known genotypes at a number of markers was part of a sample from which only allele frequencies are known. Using population genetic and statistical theory, we show that the power of such identification is, approximately, proportional to the number of independent SNPs divided by the size of the sample from which the allele frequencies are available. We quantify the limits of identification and propose likelihood and regression analysis methods for the analysis of data. We show that these methods have similar statistical properties and have more desirable properties, in terms of type-I error rate and statistical power, than test statistics suggested in the literature.

## Author Summary

It was shown recently by Homer and colleagues that it may be possible to determine whether a person with known genotypes at a number of markers was part of a pool of DNA from which only frequencies of alleles at the markers are known. In this study, we quantify how well such identification can work in practice. The larger the size of the sample from which the allele frequencies are available, the more independent genetic markers are required to allow individual identification.

## Introduction

Homer et al. [1] showed that it was possible in some circumstances to identify whether a person with observed genotypes at multiple loci was part of a sample from which only estimated allele frequencies were known. Such identification would be particularly useful in forensic science if the presence or absence of a person's DNA in a mixture of DNA could be established. The authors also discussed the relevance of their findings when summary statistics such as allele frequencies were available in the public domain as part of genotype-phenotype studies, because it possibly could be established that individuals, or their close relatives, were part of a particular study. As a result of the publication of Homer et al., NIH and the Wellcome Trust added more restrictions to the access of such data to avoid potential identifiability (http://grants.nih.gov/grants/gwas/data_sharing_policy_modifications_20080828.pdf).

The approach taken by Homer et al. was to have two samples with estimated allele frequencies, here called the “test” and “reference” sample, and to ask whether an individual was ‘close to’ either of these samples, using a statistic that measured a distance to the sample. The properties of the test statistic were not investigated theoretically (although simulation studies were performed), and the difference between “sample” and “population” was not always clear.

In this note we take a best-case idealised setting in which there is a single population from which there is a test sample with allele frequencies at a number of loci and from which there is a single individual, called the proband, with full genotypes. The question is whether the person was part of this test sample from which allele frequencies are available. We use both likelihood and linear regression theory, which illustrate different approaches to the problem, to draw inference about the hypothesis that a proband was part of the test sample. We show that the power of identification of a proband as part of a test sample is, approximately, proportional to the number of independent SNPs divided by the size of the sample from which the allele frequencies are available. The power is reduced by a predictable magnitude if the frequencies in the population are themselves estimated imprecisely. Properties of likelihood-ratios and regression test statistics and a comparison with the statistic used by Homer et al. were verified by simulation.

## Methods

### Notation and assumptions

There are *m* independent SNP markers with a population frequency of *p_i_* for allele B at the *i* ^th^ SNP. We assume Hardy-Weinberg equilibrium in the population, so that the genotype proportions for the *i* ^th^ SNP are (1−*p_i_*)^2^, 2*p_i_*(1−*p_i_*) and *p_i_* ^2^ for genotypes AA, AB and BB, respectively. We have estimated allele frequencies 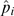 based upon a test sample of *N* unrelated individuals. In the test sample of 2*N* alleles, *n_i_* is the number of B alleles at locus *i*. In this study we assume that *N* is known and individuals are equally represented in computing 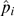. Note that these conditions are unlikely to be fully met in forensic applications when the test sample may be a DNA pool and we consider the implications later.

The genotype for proband X at the *i* ^th^ SNP is *g_i_*, which can take values of 0, 1 and 2 for genotypes AA, AB and BB, and the expectation of *y*_i_ = ½*g_i_* is the population frequency *p_i_*, *i.e.* E[½*g_i_*] = *p_i_*.

To simplify derivations, we shall first assume the population frequencies *p_i_*, are known. More generally, we assume we have prior unbiased estimates of the allele frequencies from the same population from a different finite sample (the “reference sample”) of size N*, in which there are n*_i_ B alleles at locus *i*. As both the test and reference samples are drawn independently from the population, the best estimate of the frequency in the population is given by the pooled value, 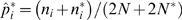 It is explained subsequently why this estimate, rather than say n*_i_/2N*, the estimate of the allele frequency from the reference sample, is used in the statistical analysis.

### Likelihood

#### Population frequencies known

If, under the assumptions described above, the numbers of individuals in the test sample and population frequencies are known, then we can compute the relative likelihood of sampling the observed genotypes under the two alternative hypotheses: the proband X is or is not in the test sample.

If X is ***not*** a member of this sample, then *n_i_*∼ Binomial(2*N*, *p_i_*) and *g_i_* is independently distributed Binomial(2, *p_i_*). Hence the joint probability of sample and proband is
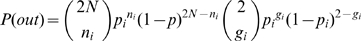
If X ***is*** a member of the sample, *n_i_* has the same distribution, but *g_i_* is sampled from the 2*N* without replacement and has the hypergeometric distribution:
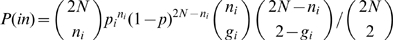
Alternatively *P*(*in*) can be viewed as *n_i_−g_i_* ∼ Binomial(2*N*−2, *p_i_*) and *g_i_* ∼ Binomial(2, *p_i_*) independently, giving the same formula.

Hence the likelihood ratio for X *in* vs *not in* (*out*) the test sample reduces to a simple equation, but in view of the varying length of the factorial expressions, it is clearer to write three separate ones:
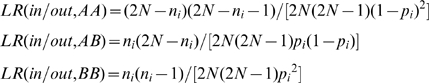
For example, if allele B is at low frequency in the population (*p_i_* small) and the proband is BB, then if the number in the sample, *n_i_*<2, LR(BB) = 0, as it should; but as *n_i_* increases LR(BB) becomes high. If the test sample is quite large, the correction for non-replacement sampling becomes less important, and the formulae simplify to, for example, *LR*(*in/out*, BB) = (*n_i_*/2*N*)^2^/*p_i_* ^2^, *i.e.* a simple comparison of whether the genotype frequencies correspond more closely to those in the sample than in the population.

For *m* independent loci, the log likelihood ratio (log*LR*) is
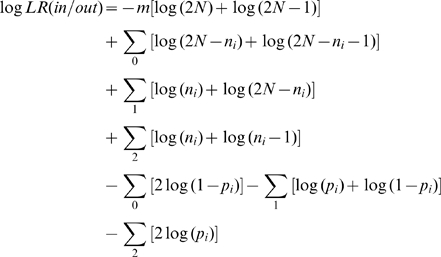
where 0, 1, 2 represent AA, AB and BB individuals at the respective loci. If the non-replacement sampling is ignored, this simplifies to a likelihood comparison of allele frequencies in an individual to one of two different populations
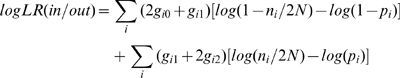
where *g_i_*_0_ etc. refer to counts over the corresponding genotypes.

#### Population frequencies estimated

If the marker frequencies are estimated from a reference sample of the population of size N*, then the allele frequencies *p_i_* in the above equations have to be replaced in the analysis by an estimate of population frequency. Although it would be possible just to use the frequencies n*_i_/2N* in the reference sample, this should *not* be done as it leads to increased expectations of log*LR* and, if unadjusted, to bias in assignment of the proband to the test sample. More appropriately, providing the reference and test samples are independent, the pooled estimate of the population frequency 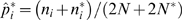 should be used instead of *p_i_* in the above formulae.

#### Properties

The likelihood ratio (or its logarithm) contains all of the information and reflects the relative probabilities of the two hypotheses (*in/out*) given the data.

We consider expectations of log*LR* under the different hypotheses. Standard statistical differentiation was employed, taking a Taylor series expansion of terms such as log(*n_i_*) about log(2*Np_i_*), ignoring higher order terms, and taking expectations over the sampling distributions of the observed frequencies under each hypothesis (see Text S1 for more details). The following formulae have also been verified by simulation.

1. If the population frequencies are known, then for a proband in the test sample, E(log*LR*|*in*)≈½*m*/*N*, and for a proband not in the test sample E(log*LR*|*out*)≈−½*m*/*N*. Therefore the ability to find whether the proband is in or not in the sample is proportional to the number of independent markers and inversely proportional to the size of the test sample.
2. The variance of log*LR* is approximately the same whether the proband is or is not present, and is close to *m*/*N* = 2E(log*LR*|in). One measure of discriminating power is the difference in expected log-likelihoods for the two hypotheses, scaled by the variance of that difference, analogous to the non-centrality parameter of a test statistic: [E(log*LR*|*in*)−E(log*LR*|*out*)]^2^/[var(log*LR*|*in*)+var(log*LR*|*out*)]≈½*m/N*. Hypothesis tests are discussed further in the subsequent section on the regression analysis, but note that the two hypothesis (*in*/*out*) are not nested. The variance under the *in* hypothesis is twice its expectation as for a chi-square with 1 degree of freedom so the proportion of *LR* exceeding some threshold can be predicted.
3. The allele frequencies have little influence on the distribution of the likelihoods. Unless the frequencies are very extreme, or the test sample very small, the expected likelihood ratios are little affected by whether the non-replacement sampling is accounted for, providing they are computable. With very small numbers of a homozygous class expected under the *out* hypothesis, then exclusions can occur with some probability. In such a case, if genotype results are correct, then presence of the proband in the test sample has to be excluded. This can occur even with relatively large test sample sizes. The joint probability of the proband having genotype AB and the test sample being homozygous AA and thereby excluded is 2*p*(1−*p*)^2*N*+1^≈2*pe* ^−*2Np*^ for small *p*, and for example is 0.0027 for *p* = 0.01 and *N* = 100.
4. If the population frequencies are estimated as 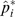, the expectations of the likelihoods and their variances and hence discriminating ability are all reduced by a proportion of approximately N*/(N+N*), *e.g.* E(log*LR*|*in*) = [N*/(*N*+N*)](½*m*/*N*). For example, the reduction is by one-half if the frequency is estimated using a reference sample of the same size as the test sample, and essentially to zero if there are no such other data.
5. If there is linkage disequilibrium amongst the loci, but the data are analysed as if they are independent, the expectation of log*LR* is the same as if all were unlinked. The sampling variances are, however, increased. If the population frequencies are known without error, it can be shown that for any pair of loci, regardless of their frequency, var(log*LR*|*in*)≈var(log*LR*|*out*)≈2(1+*r* ^2^)/*N*, approximately, where *r* ^2^ is the squared correlation of gene frequencies between these loci [2]. Hence, for *m* loci, the discriminating ability is approximately 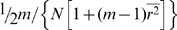 and, as the number of loci increases, asymptotes to 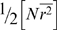, where 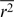 is the mean of *r* ^2^ over all pairs of loci. If this quantity can not be calculated directly it can be predicted from population parameters.

### Linear regression

We show that the main results for the regression approach are based upon the expectation that the regression of the proband frequency, *y_i_* = ½*g_i_*, on 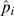, each expressed as deviations from population frequencies, is distributed about unity for all loci if the proband was part of the test sample, and about zero otherwise.

#### Population frequencies known

Considering this case first for simplicity, the regression coefficient is estimated as 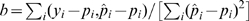. If the proband is in the test sample, *y_i_* and 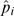 are correlated, so 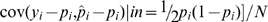, and if it is not in the test sample, 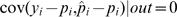. In both cases,
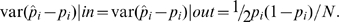
Hence, assuming many loci such that the ratio of expectations approximates the expectation of the ratios,
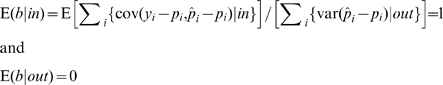
Therefore the regression of the proband's allele frequency on the estimated allele frequency in the test sample, both expressed as a deviation from the population frequency, is expected to be zero if the proband was not in the test sample and one if the probands was in the test sample. The corresponding sampling variances are, respectively, assuming large *m*,
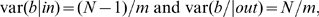

*i.e.*, the variance is slightly smaller if the proband is in the sample.

These results correspond closely to the expectations of the conditional log-likelihood analysis, and show how they are related.

#### Population frequencies estimated

There are two approaches to estimating the population frequency and testing: comparison of the proband with either the reference sample of N* alone, or comparison of the proband with the estimate 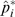 from the combined sample of size *N*+N*. Whilst it might seem counterintuitive to use the latter which includes the test data in the estimate, it provides simpler results, notably expected regression coefficients of 0 (*out*) and 1 (*in*); hence we use it here.

The estimate of the regression coefficient is 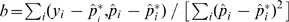. Now 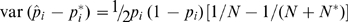. This is also 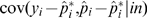, whereas 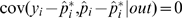. Hence, if the proband is in the test sample, 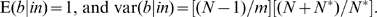
If the proband is *not in* the test sample,
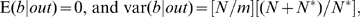
where terms of 1 relative to *N*+N* are ignored. Hence the test statistics are simply N*/(*N*+N*) of those where the population frequencies are known (*i.e.*, N*→∞).

#### Hypothesis testing

The null hypothesis is *out*, E(*b*) = 0: the proband was not part of the test sample. The alternative hypothesis (*in*, E(*b*)>0) is that the proband (or a close relative) was part of the test sample.

If hypothesis *out* is true, a test statistic for the null hypothesis that the proband is part of the sample is *t* = [*b*−1]^2^/var (*b*|*out*). Again, *t*∼χ^2^_(1)_ if this hypothesis is true. If it is false, *i.e.* the proband is not part of the sample, then *t* has a non-central chi-square distribution *t*∼χ^2′^_(1),λ_ with non-centrality λ≈(*m/N*)[N*/(*N*+N*)]. For large *N*, inferences from testing whether the proband is *in* or whether the proband is *out* of the test sample are identical, as in the likelihood approach: the probability of rejecting the null hypothesis that the proband is not part of the sample when that is false is the same as the probability of rejecting the null hypothesis that the proband is in the sample when that is false.

For a type-I error rate of α and power of 1−β, with corresponding normal deviates of z_α_ and z_1−β_, the required ratio of *m*/*N* = λ = (z_α_+z_1−β_)^2^, assuming a very large reference sample (N*≫*N*). For a type-I error rate of 0.05 and a power of 80%, the required *m*/*N* ratio is therefore approximately 6, and for α = 10^−6^ and 1−β = 99%, the ratio is approximately 50. If, for example, the reference sample were the same size as the test sample, the number of loci would have to be doubled to give the same power.

## Results

### Simulations

Population allele frequencies on *m* markers were drawn from a uniform distribution with lower bound 0.05 and upper bound 0.95 (*i.e.*, minor allele frequency (MAF)>0.05). For the *i* ^th^ SNP, a genotype score (*y*_i_) of a proband was simulated from a binomial distribution with probability *p_i_* and sample size 2. Allele frequencies in the reference and test samples were simulated from a binomial distribution with probability *p_i_* and sample size 2N^*^ and 2*N*, respectively. If the proband was part of the test sample then the test sample was simulated on *N*−1 individuals and the allele count from the proband was added to that from this sample to create a sample from *N* individuals. Linear regression was performed as described previously, for a type-I error rate of 0.05, and the Homer et al. [1] test statistic (see Text S2) was also implemented. 1000 simulations were performed for combinations of *N* = 100, 1000, 10000, N* = 100, 1000, 10000 and ∞ and *m* = 50,000, when the proband was either part or not part of the test sample.

The results are shown in Table 1. The regression type-I error rates are well controlled when the hypotheses tested are true. As predicted (Text S2), the type-I error rates for the Homer et al. test statistic are not well controlled. In many cases the probability of rejecting the null hypothesis when it is true is close to zero. Power to determine whether the proband is part of the test sample is good for test samples of 1000 if the reference sample size is large. Inference from the regression and likelihood-ratio approach is similar, as expected (Table S1).

**Table 1:** Simulation results (*m* = 50,000 SNPs; type-I error rate = 0.05; 1000 simulations).

			Linear regression			Homer et al.	
Proband in test?	N*	*N*	*b*	P(*b*>0)	P(*b*<1)}	P(*D*>0)	P(*D*<0)
				***Type-I error***	***Power***	***Type-I error***	***Power***
NO	∞	100	0.000	0.055	1.000	0.000	1.000
NO	∞	1000	0.002	0.064	1.000	0.002	0.486
NO	∞	10000	0.000	0.056	0.731	0.016	0.133
NO	100	100	0.001	0.061	1.000	0.057	0.039
NO	100	1000	0.005	0.065	0.678	0.994	0.000
NO	100	10000	0.041	0.052	0.079	0.999	0.000
NO	1000	100	−0.000	0.047	1.000	0.000	0.997
NO	1000	1000	0.014	0.069	0.999	0.060	0.047
NO	1000	10000	0.002	0.057	0.185	0.404	0.000
NO	10000	100	0.002	0.067	1.000	0.000	0.999
NO	10000	1000	0.001	0.065	1.000	0.001	0.408
NO	10000	10000	−0.002	0.053	0.472	0.048	0.051
				***Power***	***Type-I error***	***Power***	***Type-I error***
YES	∞	100	0.999	1.000	0.048	1.000	0.000
YES	∞	1000	1.003	1.000	0.051	0.996	0.000
YES	∞	10000	0.997	0.709	0.053	0.396	0.000
YES	100	100	1.004	1.000	0.064	1.000	0.000
YES	100	1000	0.999	0.686	0.060	1.000	0.000
YES	100	10000	0.974	0.078	0.063	0.998	0.000
YES	1000	100	0.999	1.000	0.058	1.000	0.000
YES	1000	1000	1.002	1.000	0.063	0.992	0.000
YES	1000	10000	1.015	0.190	0.053	0.625	0.000
YES	10000	100	1.000	1.000	0.063	1.000	0.000
YES	10000	1000	0.999	1.000	0.059	0.993	0.000
YES	10000	10000	0.998	0.475	0.067	0.375	0.000

*D* refers to the Homer et al. test statistic.

## Discussion

Simple methods were proposed to test the hypothesis of whether a proband was part of a test sample. The expected likelihood ratio or the power to reject the null hypothesis when it is false were derived and shown to be a simple function of *m/N*, the ratio of the number of markers and test sample size. If allele frequencies in the population are well-estimated then there is good power to determine if a proband is part of a sample of ∼1000 individuals when using a whole genome scan of ∼50,000 independent markers.

There is a strong relationship between the log*LR* statistic and regression test statistics. The difference in the two regression test statistics, *in* or *out* of the test sample, is approximately equal to twice the log*LR* statistic. Hence, twice the log*LR* statistic is very similar to a test statistic from regression that also tests for the *in* vs *out* hypothesis (Table S1).

Could any inference be drawn in the case where there are no prior estimates of allele frequencies? The analyses indicate that, even with many marker loci, there is little power as N* approaches 0 unless the sample size *N* is also very small, and no larger than N*.

The parameter *m* was defined as the number of independent SNPs. When many SNPs are used, *e.g.* all common SNPs on a chip, then there is correlation (linkage disequilibrium) among the SNPs. Consequently, the *y* variables (allele numbers in the proband) are correlated and not taking this into account will inflate the test statistic because the true variance of the estimated regression coefficient is larger than appears from the total number of SNPs. Similarly, the variance of the likelihood statistic is increased if allele frequencies across SNPs are correlated. There are a number of ways to deal with this correlation structure. (i) Restrict the analyses to SNPs that are in linkage equilibrium. This seems wasteful because information is discarded. (ii) Take the correlated nature of *y* into account by fitting the covariance structure of *y* into the regression or likelihood analysis. The effect of LD on the variance of the log likelihoods is shown earlier, and appropriate corrections using the mean *r* ^2^ given. In view of the correspondence of the likelihood and regression approaches, the same correction can be applied to the latter. The relevant quantity may be obtained from a separate data set (*e.g.* HapMap). (iii) Perform a theoretical adjustment on the test statistic, by calibrating the variance of the test statistic on the equivalent number of independent markers. According to population genetics theory, the number of independent loci (‘segments’) in a random population with effective size *N_e_* and genome length *L* (Morgan) is approximately 2*N_e_L*/log(4*N_e_L*) [3]. For human populations, with *N_e_* = 10,000 and *L* = 35, this implies a total of ∼50,000 SNPs. This number can also be estimated using a simulation approach, conditioning on the observed LD structure in a sample where individual-level genotype data are available. Such an application resulted in ∼55,000 independent SNPs for one genome-wide association study [4].

### Population differences

In our derivations we have assumed that all samples (proband, reference and test) are from the same population and that within the population there is random mating. What if these assumptions are violated?

If all samples are from the same population but there is deviation from HWE then the tests are somewhat biased because HWE is assumed in computing the likelihood and the variance of sample allele frequencies. Population differences are more serious and can lead to the wrong inference. There are a large number of possibilities because, in principle, the proband, reference and test samples can all come from different populations. However, population differences between the reference and test sample can be tested explicitly using standard tests for differences in gene frequency. There seems little point in testing whether a proband was part of a specific test sample when there is no reference sample from the same population. Nevertheless, what can we predict if the reference population is not actually from the same population, but is used as if it is? Then both the likelihood statistics for the hypothesis ‘*in*’ and ‘*out*’ are inflated, by essentially the same amount, so the problem is not the divergence between the two populations, but bias in the test statistic. If population frequencies are inappropriately or approximately estimated, the sample is more likely to be assigned as ‘*in*’ when it should not be. The reference sample is of little value if the divergence between the populations, expressed as Wright's *F*_ST_, approaches 1/(2*N*).

Can we quantify the limits of identification in practical situations? This is hard, because there are (at least) three difficulties in addition to the theoretical sample *m*/*N* criterion:

1. The size of reference sample used to estimate the population frequency - in effect a sort of ‘outgroup’ as *N* gets very large. So if the test sample is much larger than the reference sample (*N*≫N*) the latter provides the limit.
2. The degree to which the test *N* and the reference N* individuals are samples from the same population.
3. Linkage disequilibrium, which generates a limit regardless of numbers of loci.

For these reasons we cannot set a simple limit to identification without reference to other parameters (or speculation).

### Relatives

In the analysis we have not considered the possibility that the proband is not in the test sample, but is related to one or more persons who is. For example if a relative with relationship *R* (e.g. *R* = ½ for full sibs) is in the test sample, then the expectation of the regression coefficient is E(*b*) = *R* rather than 0 or 1. Similar calculations can be done if, for example, there are several relatives in the test or reference samples. If many markers are used, a value of *b* of approximately one-half would raise suspicions that in fact a full sib, parent or child is in the test sample. Lower, but non-zero values could be consequences of sampling or relationship. The simulation results in Table 1 illustrate how sensitive the methods can be, and hence there seems a real possibility of identifying not just the proband but also his/her relatives.

### Forensic applications

A problem frequently met in forensic applications is whether a particular individual's DNA appears in a mixture obtained at a crime scene, for example. In this case, it is usually unknown how many individuals' DNA is present in the sample (*i.e.*, *N* is unknown), equal representation cannot be assumed, and there may be allelic drop out in the sample, although Homer et al. [1] showed empirically that probands could be detected even if their contribution to the DNA pool was small. We do not therefore consider the present results to be relevant for probabilistic inference in a forensic setting. However, exclusion of a proband from a pooled DNA sample is possible if many markers are used, the actual *N* is small and frequencies of alleles from the pool are estimated accurately. The likelihood framework is sensitive to genotyping errors in that false exclusions could occur, but the analysis could be adapted to model genotype counts with specified probability of errors or by assuming replacement sampling in computing P(*in*). The linear regression approach is likely to be robust to genotyping error.

### Genome-wide association studies

In contrast to forensic applications, in the situation considered by Homer et al. in which the test sample is a database constructed using a specified number of individuals each with individual genotypes, and with the gene frequencies estimated as their average, our results support their conclusions. Probands that were part of a test sample could be identified even for samples sizes of 1000. If, for example, there are both diseased case and healthy control samples in the association test, each assumed to be sampled from the same population, then it is possible to test whether an individual is present in either the case or control group using the analysis we have described, but using each sample in turn as the test sample.

Current genome-wide association studies (and meta-analyses based upon multiple studies) are conducted on large samples, often of the order of 10,000 or so, and in this case our results show that the power to identify a proband who was part of such a large sample when the reference sample is of similar size is only about one-half (Table 1) assuming 50,000 independent loci, even under the ideal circumstances considered in this study.

## Supporting Information

Table S1 Simulation results comparing the LR and Regression statistics.
(0.06 MB DOC)

Text S1 Computation of expected likelihoods.
(0.03 MB DOC)

Text S2 Homer et al. test statistic.
(0.03 MB DOC)
