# Supplementary material for: The Limits of Individual Identification from Sample Allele Frequencies: Theory and Statistical Analysis
Source: PLoS Genet. 2009 Oct 2;5(10):e1000628. doi: 10.1371/journal.pgen.1000628 (PMC2746319; doi:10.1371/journal.pgen.1000628)
Supplement: Table S1 — Simulation results comparing the LR and Regression statistics. (0.06 MB DOC) [file pgen.1000628.s001.doc]

**Table S1**: Simulation results comparing the LR and Regression statistics.

*m* = 50,000 SNPs; *p* from Uniform(0.05, 0.95); type-I error rate = 0.05; 1000 simulations

Population frequencies known without error

|  |  |  | Likelihood (2*log(LR)) | | Linear regression (F test) | |
| --- | --- | --- | --- | --- | --- | --- |
| Proband in test? | *N** | *N* | Mean | SD | Mean | SD |
|  |  |  |  |  | ***Test b < 0*** | |
| NO^1^ | ∞ | 100 | -509.7 | 44.1 | 499.9 | 45.0 |
| NO | ∞ | 1000 | -50.2 | 14.4 | 50.9 | 15.1 |
| NO | ∞ | 10000 | -5.2 | 4.5 | 6.3 | 5.0 |
|  |  |  |  |  | ***Test b > 0*** | |
| YES | ∞ | 100 | 503.3 | 43.0 | 505.3 | 46.2 |
| YES | ∞ | 1000 | 50.2 | 14.1 | 51.5 | 15.0 |
| YES | ∞ | 10000 | 5.1 | 4.5 | 6.1 | 5.1 |

Population allele frequency estimated with error

|  |  |  | Likelihood (2*log(LR)) | | Linear regression (F test) | |
| --- | --- | --- | --- | --- | --- | --- |
| Proband in test? | *N** | *N* | Mean | SD | Mean | SD |
|  |  |  |  |  | ***Test b < 0*** | |
| NO^1^ | 100 | 100 | -257.2 | 32.7 | 248.4 | 33.0 |
| NO | 100 | 1000 | -4.6 | 4.4 | 5.6 | 4.8 |
| NO | 100 | 10000 | -0.04 | 0.4 | 1.1 | 1.6 |
| NO^1^ | 1000 | 100 | -466.9 | 41.6 | 456.0 | 42.1 |
| NO | 1000 | 1000 | -24.5 | 9.7 | 25.3 | 10.3 |
| NO | 1000 | 10000 | -0.5 | 1.3 | 1.5 | 2.0 |
| NO^1^ | 10000 | 100 | -506.5 | 46.0 | 496.9 | 46.6 |
| NO | 10000 | 1000 | -45.6 | 13.4 | 46.5 | 14.0 |
| NO | 10000 | 10000 | -2.5 | 3.2 | 3.6 | 3.7 |
|  |  |  |  |  | ***Test b > 0*** | |
| YES | 100 | 100 | 254.9 | 33.1 | 255.6 | 34.7 |
| YES | 100 | 1000 | 4.5 | 4.3 | 5.7 | 4.8 |
| YES | 100 | 10000 | 0.04 | 0.4 | 1.2 | 1.7 |
| YES | 1000 | 100 | 456.7 | 43.6 | 459.3 | 46.2 |
| YES | 1000 | 1000 | 25.2 | 9.9 | 26.2 | 10.5 |
| YES | 1000 | 10000 | 0.5 | 1.3 | 1.6 | 2.1 |
| YES | 10000 | 100 | 499.2 | 45.6 | 501.3 | 48.3 |
| YES | 10000 | 1000 | 45.5 | 13.3 | 46.5 | 14.3 |
| YES | 10000 | 10000 | 2.5 | 3.2 | 3.6 | 3.7 |

^1^ Simulation of p ~ Uniform(0.10, 0.90). For simulated population allele frequencies between 0.05 and 0.95, the likelihood ratio was –∞ for at least one SNP in at least one replicate because the test sample was homozygous for one allele and the proband had at least one of the other alleles.

Note: When there is little information to distinguish between the two hypotheses (*in*/*out*), the expected value of the LR will close to 1.0 and therefore log(*LR*) close to zero. The expected value of the F-test will be close to 1.0. This suggests the approximate relationship of F ≈ |2*log(LR)| + 1, which is consistent with the simulation results.
