# Supplementary material for: The Limits of Individual Identification from Sample Allele Frequencies: Theory and Statistical Analysis
Source: PLoS Genet. 2009 Oct 2;5(10):e1000628. doi: 10.1371/journal.pgen.1000628 (PMC2746319; doi:10.1371/journal.pgen.1000628)
Supplement: Text S1 — Computation of expected likelihoods. (0.03 MB DOC) [file pgen.1000628.s002.doc]

**Text S1: Computation of expected likelihoods**

The equation in the text for the likelihood, assuming for simplicity that the population frequencies are known and replacement sampling to obtain the proband’s genes, is

$$\log LR(in/out)=\sum_{i} (2g_{i0}+g_{i1})[\log(1-n_{i}/2N)-\log(1-p_{i})]+\sum_{i} (g_{i1}+2g_{i2})[\log(n_{i}/2N)-\log(p_{i})]$$

Taking a Taylor series expansion of, for example, log(*n_i_*) about log(2*Np_i_*) we obtain

log(*n_i_*) = log(2*Np_i_*) + [(1/*n_i_*)|*n_i_ =*2*Np_i_*)](*n_i_* – 2*Np_i_*) + ½ [-1/*n_i_*^2^*|n_i_ =*2*Np_i_*)](*n_i_* – 2*Np_i_*)^2^ + ...

so

log(*n_i_*/2*N_i_*) – log(*p_i_*) = (*n_i_* – 2*Np_i_*)/(2*Np_i_*) - ½(*n_i_* – 2*Np_i_*)2/(2*Np_i_*)^2^ + ...

E(*n*_i_) = 2*Np_i_* and E[(*n_i_* – 2*Np_i_*)^2^] = 2*Np_i_*(1 – *p_i_*).

If the proband is *not* *in* the test sample, E(*g_i_*_1_ + 2*g_i_*_2_) = 2*p_i_* independent of *n_i_* and hence

≈ -½*m*/*N*

If the proband is *in* the test sample, then for example, cov[*n_i_* (*g_i_*_1_ +2*g_i_*_2_)] = 2*Np_i_*(1 – *p_i_*), and E(log*LR*|*in*) ≈ *m/N* – ½*m*/*N* = ½*m*/*N.*

When only an estimate of gene frequency in the population is available combining the test and reference samples, $\hat{p}_{i}^{*}$= (*n_i_* + *n*_i_*)/(2*N* + 2*N**), the same basic analysis is used to compute E(log*LR*), but a Taylor expansion is taken of log(*n_i_*+*n*_i_*) about log[(2*N*  + 2*N**)*p_i_*]. Variances in the log likelihoods can be derived similarly, using just the first terms of the Taylor expansion.
