# Supplementary material for: The Limits of Individual Identification from Sample Allele Frequencies: Theory and Statistical Analysis
Source: PLoS Genet. 2009 Oct 2;5(10):e1000628. doi: 10.1371/journal.pgen.1000628 (PMC2746319; doi:10.1371/journal.pgen.1000628)
Supplement: Text S2 — Homer et al. test statistic. (0.03 MB DOC) [file pgen.1000628.s003.doc]

**Text S2: Homer et al. test statistic**

Homer et al. use a difference statistic, defined for SNP *i* as,

*D*_i_ = |*y*_i_ – $\hat{p}_{i}^{R}$| - |*y*_i_ - $\hat{p}_{i}$|

with $\hat{p}_{i}^{R}$an estimate of the allele frequency from a “reference population”. If the proband was not in the sample the authors suggest that E(*D*) approaches 0, whereas E(*D*) > 0 if the proband was part of the sample. They also mention that E(*D*) < 0 when the proband is ‘closer’ to the reference population than to the sample: “In the case of *D*(*Y_ij_*) < 0, *Y_i_* is more ancestrally similar to the reference population than to the mixture, and thus less likely to be in the mixture” (*Y_i_* is the proband in the notation of Homer et al.). The average of the *D*_i_ statistic over SNPs divided by its empirical SD is used as a test statistic.

*D* has, however, some undesirable properties. If $\hat{p}_{i}^{R}$is estimated without error ($\hat{p}_{i}^{R}$= *p*_i_), then it can be shown that the first term of *D*_i_ has an expectation of 2*p_i_*(1-*p_i_*)(1-*MAF_i_*), with *MAF_i_* the minor allele frequency of the *i*^th^ SNP. The expectation of the second term depends on the sample size (*N*). Approximately it is 0.8√[var(*y*_i_ - $\hat{p}_{i}$)], with 0.8 the mean of the absolute value of a variate which has a standard normal distribution. If the proband was part of the test sample then var(*y_i_* - $\hat{p}_{i}$) = ½*p_i_*(1-*p_i_*)(1-1/*N*). Now if we equate the two terms for *p* = *MAF*, we obtain the result that *D* is expected to be zero when *N* = 1/ [1 – 12.5*p*(1-*p*)^3^]. This property is highly undesirable because for common SNPs with allele frequencies that are estimated without error, the test statistic *D* ≈ 0 for a relatively small sample size *N* and adding SNPs does not improve discrimination. For example, when *p* = ½, *D* is close to zero for *N* = 5 (and *D* < 0 for *N* > 5). This was verified by simulation.

If the proband was not part of the test sample then the expectation of *D* is zero only if *N* and *N^*^* are identical. This is unlikely to be the case in practice, when *N* is usually smaller than *N^*^*. Consequently, E(*D*) < 0 under the null hypothesis that the proband is not in the test sample. In fact, *D* < 0 is evidence against the proband being part of the sample, even if both the proband and sample are from the same population. This is clearly seen for the hypothetical example that *N* = 1. Then $\hat{p}_{i}$is 0, ½ or 1, and |y*_i_* - $\hat{p}_{i}$| is 0 if the proband is the same individual but attains a much larger value when the proband is not the same individual. Clearly *D* will be << 0 if the proband is not part of this “sample”.
